# Modeling the Effects of Morphine-Altered Virus Specific Antibody Responses on HIV/SIV Dynamics

**DOI:** 10.1038/s41598-019-41751-8

**Published:** 2019-04-01

**Authors:** Jones M. Mutua, Alan S. Perelson, Anil Kumar, Naveen K. Vaidya

**Affiliations:** 1 Department of Computer Science, Mathematics and Physics, Missouri Western State University, St. Joseph, Missouri, USA; 2 Theoretical Biology and Biophysics Group, Los Alamos National Laboratory, Los Alamos, New Mexico USA; 3 Division of Pharmacology, School of Pharmacy, University of Missouri – Kansas City, Missouri, USA; 4 Department of Mathematics and Statistics, San Diego State University, San Diego, California USA; 5 Computational Science Research Center, San Diego State University, San Diego, California USA; 6 Viral Information Institute, San Diego State Universtiy, San Diego, California USA

## Abstract

Drugs of abuse, such as opiates, have been widely associated with enhancing HIV replication, accelerating disease progression and diminishing host-immune responses, thereby making it harder to effectively manage HIV infection. It is thus important to study the effects of drugs of abuse on HIV-infection and immune responses. Here, we develop mathematical models that incorporate the effects of morphine-altered antibody responses on HIV/SIV dynamics. Based on fitting the model to experimental data from simian immunodeficiency virus (SIV) infections in control and morphine-addicted macaques, we found that two of the most significant effects of virus specific antibodies are neutralizing viral particles and enhancing viral clearance. Using our model, we quantified how morphine alters virus-specific antibody responses, and how this alteration affects the key components of virus dynamics such as infection rate, virus clearance, viral load, CD4^+^ T cell count, and CD4^+^ T cell loss in SIV-infected macaques under conditioning with morphine. We found that in a subpopulation of SIV-infected morphine addicted macaques, the presence of drugs of abuse may cause significantly diminished antibody responses, resulting in more severe infection with increased SIV infectivity, a decreased viral clearance rate, increased viral load, and higher CD4^+^ T cell loss.

## Introduction

Human immunodeficiency virus-1 (HIV-1) continues to be one of the most endemic diseases in the world with approximately 33 million people living with the virus^1^. The frequency of use and dependence on drugs of abuse, such as opiates, among HIV-infected people is rapidly increasing with drug users constituting a large cohort among the HIV-infected population^2,3^. The Centers for Disease Control and Prevention^4^ estimated that in the US 28% of total AIDS cases and 33% of yearly new HIV cases were associated with the use of drugs of abuse. These statistics reflect that the use of drugs of abuse drastically exacerbates the public health burden. More importantly, drug users, once infected with HIV, are at a greater risk of suffering from higher viral load, rapid disease progression, and higher HIV-associated neurocognitive disorders (HAND)^5–7^. It is thus critical to understand how drugs of abuse affect the viral dynamics within HIV-infected individuals.

One of the effects of drugs of abuse that has been shown in laboratory experiments is the alteration of virus-specific antibody responses^8^. Virus-specific antibodies have a significant role of protecting individuals against a wide variety of viral infections. These antibodies are known to play a role in controlling established HIV infection, and preventing new infections^9^. These observations indicate that the drugs of abuse can impact virus dynamics indirectly by altering antibody responses. Therefore, when devising antibody mediated controls, such as with vaccines, it is important to study how the alteration of antibody responses due to the presence of drugs of abuse can change various aspects of viral dynamics.

Experiments utilizing simian immunodeficiency virus (SIV) infection in morphine addicted macaques have provided useful understanding of antibody responses in the presence of drugs of abuse^8^. While morphine addicted animals show antibody responses of relatively smaller magnitude than control animals^8^, whether these differences are significant in noticeably changing viral infection dynamics is not yet understood. Experimental evidence and the recent modeling study^10^ indicate that morphine use does not seem to significantly affect immune responses during the first 12 weeks post-infection. However, antibody responses and the effects of morphine on antibody levels become significantly pronounced over a longer period of time post-infection^8^. Moreover, careful consideration of the longer-term data obtained from individual SIV-infected animals indicated that about half of the morphine addicted animals studied exhibited rapid disease progression resulting in a very short lifespan^8^. This suggests that as far as the effects of morphine on long-term SIV infection is concerned, there are two different subpopulations of morphine-addicted animals, namely, a rapid-progressor morphine group and a slow-progressor morphine group as categorized in Kumar *et al*.^8^. Aligned with these different responses of animals to SIV infection under morphine conditioning, the rapid-progressor morphine group did not develop detectable antibody responses, whereas the slow-progressor morphine group and the control group did. Thus, there appears to be a complex relationship among morphine, antibody responses and virus dynamics that modeling may be able to reveal.

Mathematical modeling of virus dynamics has been useful in understanding the interplay of viral dynamics and immune responses^11–17^. Here, we develop viral dynamic models that incorporate virus-specific antibody responses to study the quantitative effects of morphine-altered antibody responses on HIV/SIV infection dynamics over the first 200 days post infection. The models are parameterized using viral load and virus-specific antibody data from morphine-addicted macaques infected with a mixture of SIV and SHIV (simian human immunodeficiency virus). Using the best model obtained from the data fitting process, we examine how morphine-altered antibody responses affect viral infectivity, viral clearance, the basic reproduction number, viral load, and CD4^+^ T cell count in each of three groups of SIV infected macaques, rapid-progressor morphine, slow-progressor morphine, and control groups.

## Materials and Methods

### Ethics Statement

Rhesus macaques used for the study were obtained from the Caribbean Research Primate Center and housed in the Animal Facility, approved by Association for Assessment and Accreditation of Laboratory Animal Care International, at the University of Puerto Rico Medical School, San Juan. The experimental protocol was approved by the Institutional Animal Care and Use Committee, and the research was performed in accordance with the Guide for the Care and Use of Laboratory Animals.

### Experiment and Data

The data used in this study was obtained from an experiment involving 12 male rhesus macaques (*Macacamulatta*) – six morphine-dependent and six control macaques^8,18^. The animals were negative for simian T-cell leukemia virus type 1 and simian retrovirus. The morphine dependence was established by injecting intramuscularly increasing doses of morphine (1–5 mg/kg) over a 2-week period. All 12 animals were infected intravenously with mixture of viruses *SHIV*_*KU*−1*B*_, *SHIV*_89−6*P*_, and *SIV*_17*E*−*Fr*_. These animals were monitored for a period of 28 weeks, and levels of circulating CD4^+^ T cells and viral loads were measured at weeks 0, 1, 2, 3, 4, 6, 8, 10, 12, 14, 16, 18, 20, 22, 24, 26, and 28 post-infection, and virus-specific neutralizing antibodies were measured once every 4 or 8 weeks post-infection. CD4+ T cells, viral loads, and virus-specific antibodies were measured using flow cytometry, real time RT-PCR, and ELISA techniques, respectively^8^. The experiment is described in more detail in Kumar *et al*.^8^. The morphine-dependent animals were maintained on morphine throughout the study period.

### Mathematical model

**Figure 1:**
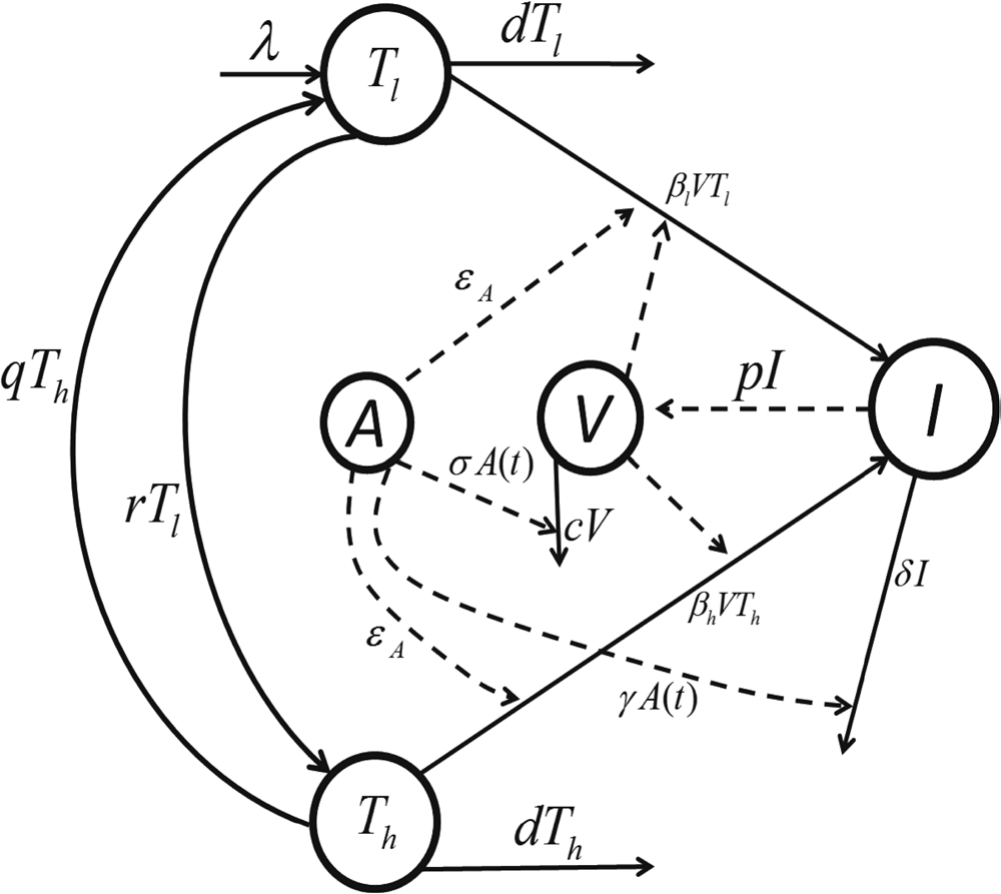
Schematics diagram of the model. Uninfected CD4^+^ T cells: *T*_*l*_ (target cells with lower susceptibility to infection) and *T*_*h*_ (target cells with higher susceptibility to infection); infected cells: *I*; free virus: *V*; virus-specific antibody responses: *A*; target cells generation rate: *λ*; death rate of uninfected cells: *d*; infection rates: *β*_*l*_ and *β*_*h*_; infected cells death rate: *δ*; virus production rate: *p*; virus clearance rate: *c*; transition rates: *r* and *q* from *T*_*l*_ to *T*_*h*_ and *T*_*h*_ to *T*_*l*_, respectively; efficacy of virus neutralization: *ε*_*A*_; enhanced virus clearance rate: *σA*(*t*); and destruction of infected cells rate: *γA*(*t*).

The model used in this study generalizes the viral dynamics model previously used to describe HIV infection in humans and SIV infection in macaques^10,19–23^. In particular, we extend a previous SIV dynamics model under morphine conditioning^10^ by incorporating the effects of virus-specific antibody responses. In a previous study^10^, we modeled the effects of morphine seen early in infection, i.e., within 3 months post infection, where immune responses were largely absent. Here we focus on modeling the effects of morphine for a longer period of time, during which virus-specific antibody responses become important. The schematic diagram of the model is presented in Fig. 1. As in Vaidya *et al*.^10^, the model consists of two subpopulations of target cells (CD4^+^ T cells), *T*_*l*_ (target cells with lower susceptibility to infection) and *T*_*h*_ (target cells with higher susceptibility to infection), categorized based on the level of co-receptor expression, which as we previously showed could be affected by morphine conditioning^10^. In addition, the model contains productively infected cells, *I*, and free virus, *V*. We assume that target cells are generated at a constant rate *λ* and die at per capita rate *d*. Upon interaction with free virus, target cells, *T*_*l*_ and *T*_*h*_, become infected at rates *β*_*l*_ and *β*_*h*_, respectively. Infected cells die at a per capita rate *δ* and produce virus at a rate *p* per infected cell. Virions are cleared at per capita rate *c*. The parameters *r* and *q* denote the transition rates from*T*_*l*_ to *T*_*h*_ and *T*_*h*_ to *T*_*l*_, respectively.

As in Tomaras *et al*.^24^, we consider three major possible effects of virus-specific antibodies: reduction in virus infectivity, i.e. virus neutralization, with efficacy *ε*_*A*_, enhanced virus clearance due to antibody binding to cell-free virus with per capita rate *σA*(*t*), and antibody-dependent destruction of infected cells with per capita rate *γA*(*t*). Here, *A*(*t*) represents the time course of virus-specific antibody levels. We model the efficacy of virus neutralization by antibody using the formula ${\varepsilon }_{A}=\frac{\eta A(t)}{1+\eta A(t)}$, whose value lies between 0 and 1 with *ε*_*A*_ = 0 in the absence of antibody (i.e., *A*(*t*) = 0) and *ε*_*A*_ = 1 for extremely high antibody levels (i.e. *A*(*t*) → ∞). *η*, *σ* and *γ* are constants introduced to represent the net effect of antibodies on the virus dynamics parameters governing viral infection, viral clearance and infected cell death, respectively. Note that *η* = 0, *σ* = 0 and *γ* = 0 represent the model corresponding to the absence of antibodies^24^. Virus-specific antibody data^8^ show that following infection the virus-specific antibody level remains low, then gradually increases and finally saturates to a maximum level (see Supplementary Fig. S1). To capture this trend, we model the antibody response curve as $A(t)=\frac{a{t}^{n}}{{b}^{n}+{t}^{n}}$, where *a* represents the maximum antibody level, *b *represents the time post-infection when the antibody level becomes half of the maximum and *n* is a Hill coefficient. The full model we study is described by the following set of equations:

1 $$\begin{array}{cc}\frac{d{T}_{l}}{dt}=\lambda +q{T}_{h}-d{T}_{l}-r{T}_{l}-(1-{\epsilon }_{A}){\beta }_{l}V{T}_{l}, & {T}_{l}(0)={T}_{l0},\\ \frac{d{T}_{h}}{dt}=r{T}_{l}-q{T}_{h}-d{T}_{h}-(1-{\epsilon }_{A}){\beta }_{h}V{T}_{h}, & {T}_{h}(0)={T}_{h0},\\ \frac{dI}{dt}=(1-{\epsilon }_{A}){\beta }_{l}V{T}_{l}+(1-{\epsilon }_{A}){\beta }_{h}V{T}_{h}-\delta I-\gamma A(t)I, & I(0)={I}_{0},\\ \frac{dV}{dt}=pI-cV-\sigma A(t)V, & V(0)={V}_{0},\end{array}$$

where

2 $$A(t)=\frac{a{t}^{n}}{{b}^{n}+{t}^{n}}$$

and

3 $${\varepsilon }_{A}=\frac{\eta A(t)}{1+\eta A(t)}.$$

### Model comparison

To determine which effects of antibodies play an important role in virus dynamics, we fit different variants of model (1) to the viral load data. The models were compared using the Akaike’s Information Criterion (AIC), which is given by the following formula^25^.

4 $$AIC=M\,{\mathrm{log}}_{e}(\frac{J}{M})+\frac{2M({N}_{p}+1)}{M-{N}_{p}-2},$$

where *J* is the sum of squared residuals, *M* is the number of data points used in the fitting, and *N*_*p*_ is the number of parameters estimated in each case.

### Parameter estimation and data fitting

As discussed in Vaidya *et al*.^10^, we take *T*_*h*0_ = 40,980/ml, *T*_*l*0_ = *T*(0) − *T*_*h*0_ for the control group, and *T*_*h*0_ = 60,650/ml, *T*_*l*0_ = *T*(0) − *T*_*h*0_ for the morphine groups (both rapid-progressor and slow-progressor) as the initial populations of target cells with lower susceptibility to infection (*T*_*l*_) and with higher susceptibility to infection (*T*_*h*_), where *T*(0) is the total number of initial target cells per ml. According to the estimate of the number of target cells for SIV infection in macaques^26^, we take 5% of the measured CD4 count as the value of *T*(0). As estimated in Mohri *et al*.^27^ and Stafford *et al*.^23^, we take 100 days as the average life span of uninfected target cells, i.e., *d* = 0.01 per day. Since the animals were initially uninfected, we set *I*_0_ = 0^26^. As estimated previously^10^, we take the virus infectivity rates as *β*_*l*_ = 5.72 × 10^−10^/RNA/day in the case where they are fixed and *β*_*h*_ = 100 × *β*_*l*_. Chen *et al*.^28^ estimated the SIV burst size *in vivo* in rhesus macaques as approximately 5 × 10^4^ virions per infected cell. Because productively infected cells live about 1 day^29^, we take the viral production rate *p* = 5 × 10^4^ virions per day per infected cell. As estimated by Ramratnam *et al*.^30^, the virion clearance rate during chronic infection in humans varies between 9.1 per day and 36 per day. Here we use the average *c* = 23 per day. However, we recognize that this rate might be higher in macaques^31^.

Each macaque was infected intravenously with 2-ml inoculums containing 10^4^ TCID_50_ of each of *SHIV*_*KU*−1*B*_, *SHIV*_89−6*P*_, and *SIV*_17*E*−*Fr*_^8^. The total of 3 × 10^4^ TCID_50_ of viruses comprises at least 3 × 10^5^ SIV RNA copies^32^. A macaque, on average, weighs 1/10 of a human, which approximately gives 1.5 liters of extracellular water and 300 ml of blood plasma in a macaque. For our base case, we assumed that the infused virions (RNA copies) are dispersed into the extracellular water. In this case, the initial viral load, *V*_0_, can be estimated as *V*_0_ ≈ 3 × 10^5^/1.5 L ≈ 200 viral RNA copies/ml, and thus, we take *V*_0_ = 200 copies/ml for base case computation. However, we also estimated parameters assuming a possible case in which the infused virions are dispersed in the 300 ml of blood plasma, i.e, *V*_0_ ≈ 1000 viral RNA copies/ml (Supplementary Table S2). We did not find significant difference in estimated parameters between whether the initial viral RNA copies are dispersed in extracellular water or in blood.

We estimated the parameters *a*, *b*, and *n *of the antibody response curve, *A*(*t*), by fitting the curve to the virus-specific antibody data using the nonlinear least-squares “lsqcurvefit” solver in MATLAB. We then used *A*(*t*) as a known function in the viral dynamics model. We solved the system of ordinary differential equations (ODEs) numerically using the “ode15s” solver in MATLAB to estimate *λ*, *q*, *r* and the immune-reponse related parameters. The predicted log_10_ viral load values were fitted to the corresponding log-transformed viral load data using nonlinear least squares regression, in which the sum of the square residuals, i.e., the difference between the model predictions and the corresponding experimental data values, is minimized. The following formula was used for calculating the sum of the squared residuals:

5 $$J=\frac{1}{M}{\sum }_{i=1}^{M}{(\mathrm{log}V({t}_{i})-\mathrm{log}\bar{V}({t}_{i}))}^{2}$$

where *M* represents the total number of data points considered for fitting, and *V* and $\bar{V}$ represent the virus concentrations predicted by the model and those given by the experimental data, respectively. For each best-fit parameter estimate, we provide 95% confidence intervals (CI), which were computed from 500 replicates, by bootstrapping the residuals^33,34^. Unless otherwise stated, we use a two-tailed test with two samples of unequal variance to test for significance of the estimated parameters in this study.

## Results

### Morphine-altered virus-specific antibodies

**Figure 2:**
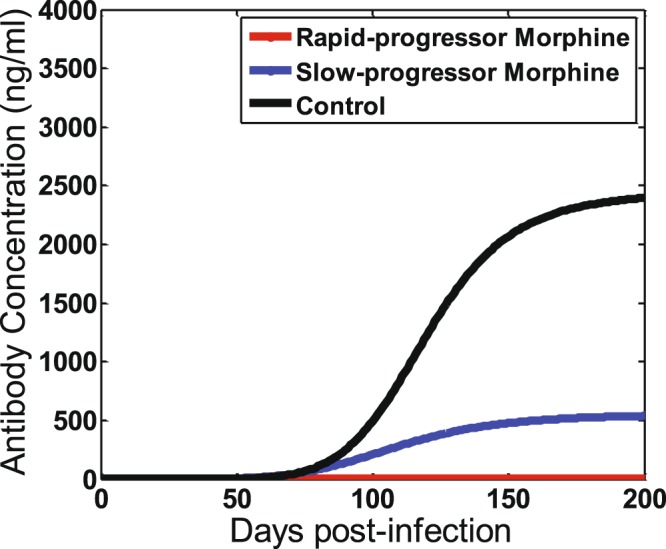
Virus-specific antibody curve plotted using median values of *a*, *b*, and *n*.

Using experimental data, we obtained the antibody response curve, *A*(*t*), for each animal from the rapid-progressor morphine group, the slow-progressor morphine group, and the control group. The data for the rapid-progressor morphine group shows that HIV-specific antibody level remains below the detection limit throughout the lifetime of these animals. Therefore, to describe a reasonable antibody response curve for this group, we take *b* = 0, which implies *A*(*t*) = *a*. The value of *a* is taken as half of the detection limit for the rapid-progessor group. The estimated values for *a*, *b*, and *n* for all animals along with their median values are given in Table 1. The best-fit curves for each animal are shown in Supplementary Fig. S1. To highlight the distinction between the groups, we also plotted the curves for each group corresponding to median values of *a*, *b*, and *n* (Fig. 2). As mentioned earlier, the pattern of antibody response is that initially the antibody level remains relatively low, then gradually increases and later saturates. While we acknowledge that we have been able to observe long-term pattern (beyond 200 days) of antibody data in only 3 out of the 12 animals (Fig. S1), the similar pattern of antibody levels saturating in a long run has also been revealed in experimental data from HIV infected patients^35^. Our estimates show that the maximum antibody level, *a*, and the time post-infection when the antibody level becomes half of the maximum, *b*, in the rapid-progressor morphine group are significantly lower (p < 0.05) than the control group (median *a* = 0.5 ng/ml*, b* = 0 days for the rapid-progressor morphine group versus the median *a* = 2444 ng/ml, *b* = 119.9 days for the control group) (Table 1, Fig. 2).

**Table 1:** Estimated values for *a*, *b*, and *n* for individual animals, and p-values used to test significance of the estimated values.

Animal	*a* (*ng*/*ml*)	*b*(*days*)	*n*
**Rapid-progressor morphine group**			
1/04L	0.50	0	Not applicable
1/28Q	0.50	0	Not applicable
1/42N	0.50	0	Not applicable
Median	0.50	0	Not appliacble
**Slow-progressor morphine group**			
1/52N	6264	223.60	2.20
1/56L	557	109.30	6.99
1/02N	256	92.00	5.66
Median	557	109.30	5.66
**Control group**			
2/31P	3128	118.50	10.50
2/02P	2028	121.60	4.70
2/AC42	1359	127.90	13.20
MAC-1	2860	81.40	3.00
MAC-2	2026	56.90	11.00
MAC-3	3800	121.30	1.40
Median	2444	119.90	7.60
**p-value**			
Rapid vs. Slow-progressor morphine group	0.3509	0.0755	Not applicable
Slow-progressor morphine vs. control group	0.9376	0.4678	0.3697
Rapid-progressor morphine vs. control group	0.0009	0.0003	Not applicable

When we compared the slow-progressor morphine group with the control group, we found that none of the antibody curve related parameters are significantly different (Table 1), showing that in the slow-progressor morphine group morphine has minimal effect on the measured antibody responses. However, we note that animal “1/52N” has an extremely high estimated value, *a* = 6264 ng/ml, while all other animals have a value of *a* less than 3800 ng/ml. Also, the set point CD4 count of this animal remains extremely high throughout the infection; its set point CD4 count is higher than 700 cells/μL^8^, while the maximum set point CD4 count of all other animals in the morphine group is 39 cells/μL^8^. Excluding animal 1/52N, the value of *a* in the slow-progressor morphine group is significantly lower than that in the control group (p < 0.05).

Between the two morphine groups (rapid-progressor and slow-progressor), none of the antibody curve related parameters are significantly different (Table 1). Again, excluding animal “1/52N”, and using two sample t-test with equal but unknown variances, *a* and *b* become significantly different (p < 0.05) between the two groups of animals under morphine conditioning. While the effects of morphine on altering antibody responses can be quite variable among animals, the antibody responses can be severely hampered in some animals due to the presence of morphine.

### Viral dynamics model selection

To identify the important model components representing the effect of morphine-altered antibody responses on explaining viral dynamics, we considered 7 different variants of model (1) in which we leave out one or more hypothesized effects of virus-specific antibody on viral neutralization, enhanced virion clearance or antibody-mediated cellular loss and then compared them based on the AIC values of the best fit to the median viral load data for the first 28 weeks post-infection (Table 2). The antibody response curve *A*(*t*) presented earlier was used in each of these models. For a fair comparison of models, we made six parameters free in each case. In each model, we estimated the same set of parameters that represent the certain effect on the viral dynamics model, regardless of whether the virus-specific antibody is assumed to alter that particular effect or not. For example, we estimated the net infection rate of lower susceptible cells in each case. The net infection rate in our model is given by $(1-{\epsilon }_{{\rm{A}}}){{\rm{\beta }}}_{{\rm{l}}}=(1-\frac{{\rm{\eta }}A(t)}{1+{\rm{\eta }}A({\rm{t}})}){{\rm{\beta }}}_{{\rm{l}}}=(\frac{1}{1+{\rm{\eta }}A(t)}){{\rm{\beta }}}_{{\rm{l}}}$. Therefore, we estimated this net effect in each case through the parameter η if η > 0 (alteration by antibody) or through the parameter β_l_ if η = 0 (no alteration by antibody) (see Table 2). The similar process was used for the estimates of effects of antibody on viral clearance and infected cell death. Note that even in the cases when the parameters β_l_, δ, and c were fixed, there is a variability in infection rate, virus clearance rate, and infected cell death rate given by the estimates of η, γ, and σ, respectively.

**Table 2:** Fitted parameters, calculated Akaike’s Information Criterion (AIC) and Sum of Squared Residuals (SSR) for model fits to median data for each model.

Model	Fitted parameters	Rapid-progressor morphine group		Slow-progressor morphine group		Control group	
		SSR	AIC	SSR	AIC	SSR	AIC
Model – 1 (Basic model)	*η*, *γ*, *σ*, *λ*, *r*, *q*	8.30	125.3	3.37	7.6	2.65	9.4
Model – 2 (*γ* = 0)	*η*, *δ*, *σ*, *λ*, *r*, *q*	6.17	122.6	3.13	6.5	2.38	7.9
Model – 3 (*η* = 0)	*β*_*l*_, *γ*, *σ*, *λ*, *r*, *q*	8.28	125.3	3.36	7.6	4.02	15.2
Model – 4 (*σ* = 0)	*η*, *γ*, *c*, *λ*, *r*, *q*	7.46	124.3	3.27	7.1	2.87	10.3
Model – 5 (*γ* = *η* = 0)	*β*_*l*_, *δ*, *σ*, *λ*, *r*, *q*	8.37	125.3	3.19	7.0	2.42	8.2
Model – 6 (*γ* = *σ* = 0)	*η*, *δ*, *c*, *λ*, *r*, *q*	8.08	125.0	3.38	7.7	2.45	8.3
Model – 7 (*σ* = *η* = 0)	*β*_*l*_, *γ*, *c*, *λ*, *r*, *q*	8.18	125.1	3.26	7.1	2.78	10.1
Model – 8 (*σ* = *η* = *γ* = 0)	*β*_*l*_, *δ*, *c*, *λ*, *r*, *q*	8.09	125.0	3.69	9.0	5.21	18.8

The parameters fitted in each model are listed in Table 2. Model-2 that only incorporates the effect of antibodies on virus neutralization and enhanced virus clearance had the lowest AIC (Table 2). However, other models, e.g., models 4, 5 and 6 also had low values of AIC. We further examined in detail whether Model-2 was the best one by fitting individual animal data to the full model, i.e. model-1. In this case, we obtained extremely small values of *γ* (on the order of 10^−9^) in most of the animals (Supplementary Table S1), asserting that there is almost no effect of antibodies on infected cell killing. We also performed the fittings for *A*(*t*) using a spline curve fit to the virus-specific antibody response in each animal and found that the fitting was not improved. In addition, the explicit formula for *A*(*t*) (Eq. (2)) allows clear comparison between the different groups. Therefore, we use *A*(*t*) given by Eq. (2) and model-2, which has the lowest AIC value, to provide further results on morphine-altered antibody responses in the sections below.

### Variation of parameter estimates among animals in three groups

**Figure 3:**
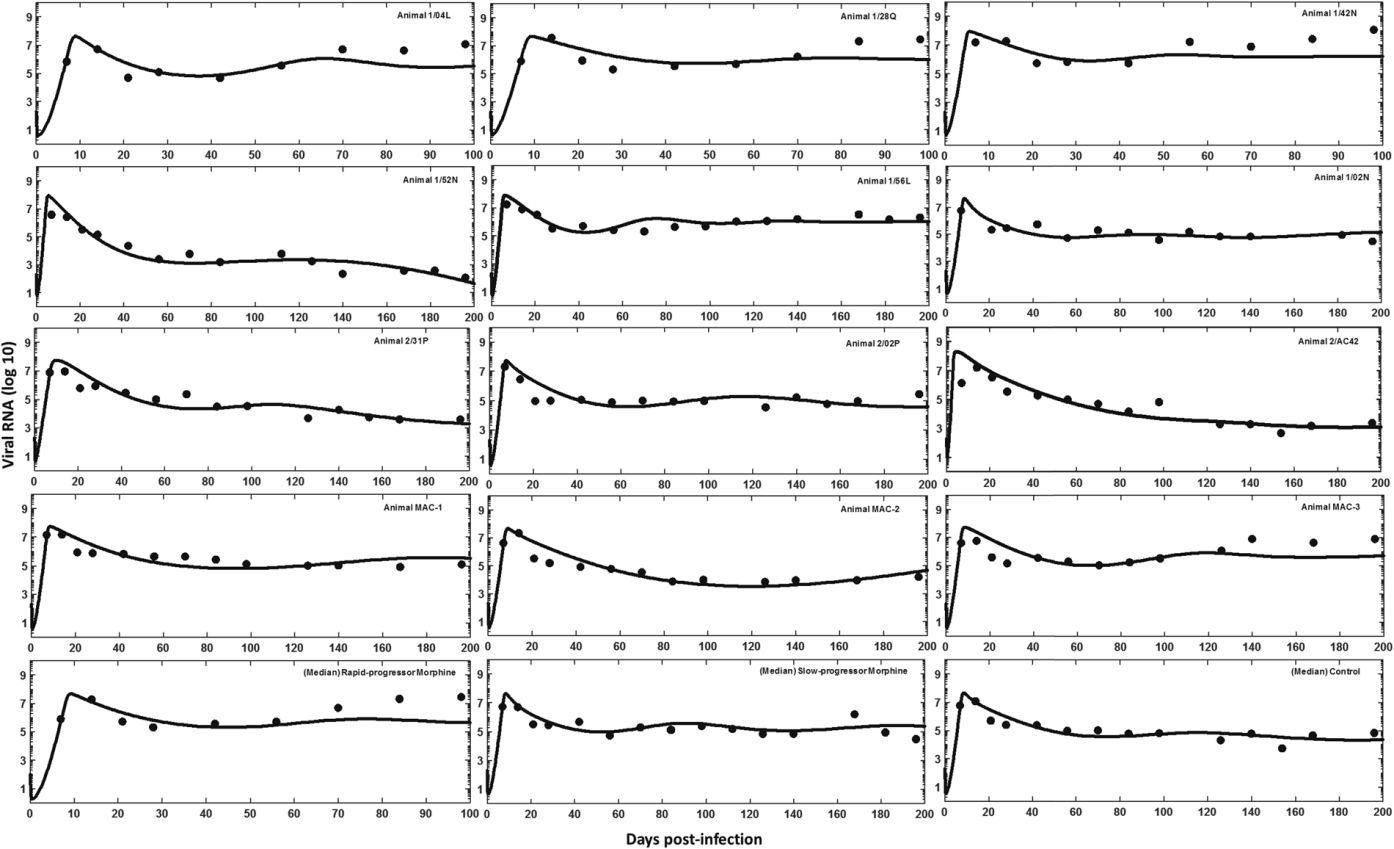
Best-fit viral dynamics curve using model-2 (solid line) to the experimental viral load data (•) for individual animals (rapid-progressor morphine group: 1/04L, 1/28Q, 1/42N; slow-progressor morphine group: 1/52N, 1/56L. 1/02N; control group: 2/31P, 2/02P, 2/AC42. MAC-1, MAC-2, MAC-3), and to the median viral load data for the rapid-progressor morphine group, the slow-progressor morphine group, and the control group.

Using the antibody response curve *A*(*t*) (Eq. 2) as a known function in model-2, we estimated the parameters by fitting the viral load data from the individual animals in the rapid-progressor morphine, the slow-progressor morphine and the control groups. These parameter estimates along with the 95% bootstrap confidence intervals of the estimated parameters are given in Table 3. The corresponding best-fits to the data from each animal and the median data of each of the three groups of animals are shown in Fig. 3. Among the estimated parameters, we found that the scaling factor associated with the effect of virus-specific antibody responses on virus neutralization, *η*, and the transition rate from *T*_*h*_ (target cells with higher susceptibility to infection) to*T*_*l*_ (target cells with lower susceptibility to infection), *q*, are significantly different between the rapid-progressor and the slow-progressor morphine groups, and between the rapid-progressor morphine and the control groups (p < 0.05). This observation is consistent with results discussed in Vaidya *et al*.^10^. Similarly, as in the previous study^10^, the estimated parameter values for the transition rate from *T*_*l*_ (target cells with lower susceptibility to infection) to *T*_*h*_ (target cells with higher susceptibility to infection), *r*, are higher in the morphine-dependent groups than in the control group. However, this difference was not statistically significant (p > 0.05). This could be because of the smaller number of animals in the two morphine groups, as the morphine treated animals were subdivided into two groups in this study and/or because of consideration of our model over a longer period of time during which virus-specific antibodies play important roles in virus dynamics (Fig. 2). In addition, we did not observe any significant difference in the other estimated parameters (p > 0.05) among the groups. As shown by our median data best-fit curves (Fig. 3), we observe a higher set-point viral load in the rapid-progressor and in the slow-progressor morphine groups than in the control group (5.4 log_10_ in the rapid-progressor morphine group, 5.2 log_10_ in the slow-progressor morphine group, and 4.2 log_10_ in the control group). This observation is consistent with the experimental results in Kumar *et al*.^8^.

**Table 3:** Model-2 (the best model) estimated parameters for individual animals, fitted parameter values to the group median data (the median data of each group), and their 95% confidence intervals in parentheses, and the mean values of *ε*_*A*_ calculated over a period of 200 days post infection using $\overline{{\varepsilon }_{A}}=\frac{1}{200}{\int }_{0}^{200}(\frac{\eta A(t)}{1+\eta A(t)})dt$. For the rapid-progressor morphine group, we can obtain the analytic solution for $\overline{{\varepsilon }_{A}}=\frac{\eta a}{1+\eta a}$.

Animal	*λ* (*cell ml*^−1^*day*^−1^)	*r* (*day*^−1^)	*q* (*day*^−1^)	*δ* (*day*^−1^)	*σ* (*ml ng*^−1^*day*^−1^)	*η* (*ml ng*^−1^)	mean value $\overline{{{\boldsymbol{\varepsilon }}}_{{\boldsymbol{A}}}}$
**Rapid-progressor morphine group**							
1/04L	3630 (3396–3901)	0.20 (0.14– 0.28)	1.1 × 10^−4^ (1.5 × 10^−5^–2.1 × 10^−4^)	0.56 (0.43–0.75)	2.8 × 10^−3^ (6.8 × 10^−4^–5.1 × 10^−3^)	1.0 × 10^−4^ (1.8 × 10^−6^–1.9 × 10^−4^)	5.0 × 10^−5^
1/28Q	3773 (3322–4481)	0.24 (0.18–0.48)	1.0 × 10^−4^ (2.6 × 10^−5^–1.4 × 10^−4^)	0.80 (0.68–0.94)	2.3 × 10^−3^ (8.0 × 10^−5^–3.0 × 10^−3^)	1.1 × 10^−4^ (1.9 × 10^−8^–1.2 × 10^−4^)	5.0 × 10^−5^
1/42N	5000 (4688–5278)	0.40 (0.16–0.69)	1.2 × 10^−4^ (8.5 × 10^−6^–2.5 × 10^−4^)	0.30 (0.26–0.53)	2.3 × 10^−3^ (2.3 × 10^−4^–4.6 × 10^−3^)	1.1 × 10^−4^ (2.8 × 10^−6^–1.9 × 10^−4^)	5.0 × 10^−5^
Group median data	3630 (3440–3835)	0.16 (0.06–0.34)	1.0 × 10^−2^ (1.4 × 10^−5^–2.0 × 10^−2^)	0.31 (0.09–0.47)	1.1 × 10^−2^ (7.4 × 10^−3^–1.4 × 10^−2^)	1.9 × 10^−6^ (4.1 × 10^−7^–3.3 × 10^−6^)	9.5 × 10^−7^
**Slow-progressor morphine group**							
1/52N	3631 (3629–3632)	0.38 (0.31–0.47)	0.21 (0.08–0.35)	0.53 (0.44–0.63)	1.5 × 10^−2^ (1.2 × 10^−2^–1.8 × 10^−2^)	1.0 × 10^−6^ (1.3 × 10^−7^–2.0×10^−6^)	1.1 × 10^−3^
1/56L	3629 (3627–3631)	0.41 (0.28–0.62)	0.18 (0.04–0.34)	0.32 (0.24–0.36)	2.3 × 10^−6^ (1.5 × 10^−7^–4.4×10^−6^)	1.0 × 10^−6^ (6.8 × 10^−8^–1.8 × 10^−6^)	2.4 × 10^−4^
1/02N	3630 (3511–3757)	0.18 (0.15–0.22)	0.10 (0.02–0.18)	0.75 (0.55–0.96)	3.0 × 10^−2^ (1.2 × 10^−2^–4.8 × 10^−2^)	1.1 × 10^−6^ (2.6 × 10^−8^–1.9 × 10^−6^)	1.5 × 10^−4^
Group median data	3629 (3627–3630)	0.17 (0.11–0.22)	0.10 (0.02–0.21)	0.65 (0.42–0.68)	3.4 × 10^−3^ (1.0 × 10^−3^–5.5 × 10^−3^)	1.0 × 10^−6^ (3.3 × 10^−7^–2.6 × 10^−6^)	2.4 × 10^−4^
**Control group**							
2/31P	3629 (3593–3661)	0.31 (0.23–0.41)	0.68 (0.51–0.91)	0.31 (0.24–0.34)	7.1 × 10^−3^ (5.5 × 10^−3^–1.0×10^−2^)	1.0 × 10^−6^ (1.1 × 10^−7^–2.0 × 10^−6^)	1.2 × 10^−3^
2/02P	4050 (3969–4125)	0.18 (0.13–0.24)	0.20 (0.10–0.34)	0.66 (0.40–0.74)	2.3 × 10^−3^ (7.1 × 10^−4^–4.4 × 10^−3^)	1.0 × 10^−4^ (3.7 × 10^−5^–2.0 × 10^−4^)	6.6 × 10^−2^
2/AC42	3630 (3627–3632)	0.14 (0.11–0.18)	0.33 (0.14–0.46)	0.54 (0.46–0.61)	5.3 × 10^−4^ (2.8 × 10^−4^–3.0 × 10^−3^)	4.9 × 10^−6^ (1.2 × 10^−7^–1.9 × 10^−5^)	2.2 × 10^−2^
MAC-1	3630 (3115–3893)	0.13 (0.10–0.25)	0.18 (0.07–0.29)	0.38 (0.16–0.39)	2.3 × 10^−3^ (3.0 × 10^−4^–3.4×10^−3^)	1.0 × 10^−4^ (1.4 × 10^−5^–1.5 × 10^−4^)	1.3 × 10^−1^
MAC-2	3629 (3610–3647)	0.13 (0.10–0.16)	0.28 (0.12–0.45)	0.61 (0.40–0.82)	3.2 × 10^−6^ (7.1×10^−7^–5.9 × 10^−6^)	1.0 × 10^−7^ (9.9 × 10^−9^–2.0 × 10^−6^)	1.4 × 10^−4^
MAC-3	3630 (3574–3684)	0.16 (0.08–0.29)	0.24 (0.06–0.43)	0.40 (0.24–0.53)	2.3 × 10^−6^ (1.4 × 10^−7^–4.6 × 10^−6^)	1.0 × 10^−6^ (5.9 × 10^−8^–2.0 × 10^−6^)	1.5 × 10^−3^
Group median data	3630 (3628–3631)	0.15 (0.12–0.19)	0.18 (0.07–0.31)	0.65 (0.41–0.81)	2.5 × 10^−3^ (1.1 × 10^−3^–4.4 × 10^−3^)	1.1 × 10^−6^ (2.2 × 10^−7^–2.7 × 10^−6^)	1.0 × 10^−3^

### Effects of morphine-altered antibody responses on virus neutralization and enhanced viral clearance

**Figure 4:**
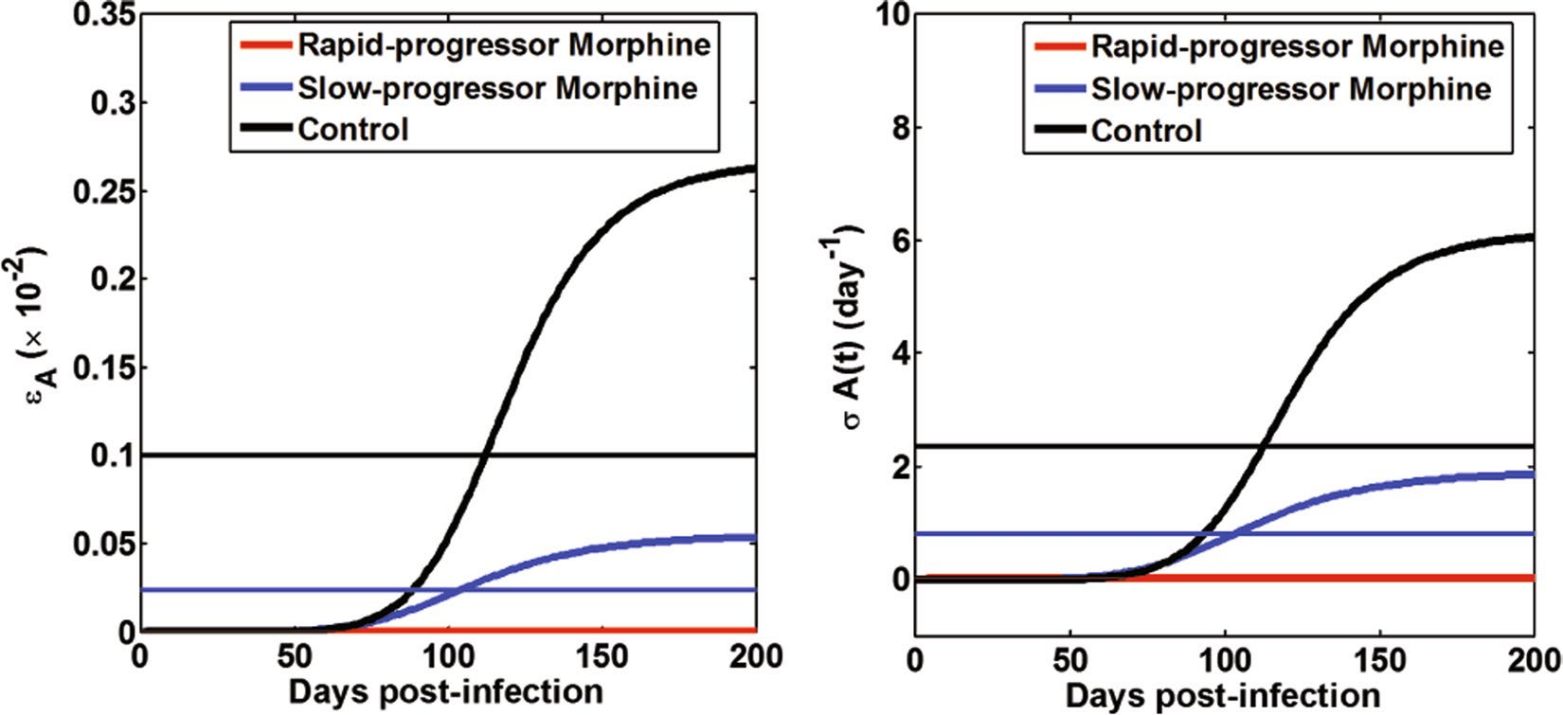
Predicted effects of morphine-altered virus-specific antibody responses on virus neutralization and enhanced virus clearance. The horizontal lines show the mean values of *ε*_*A*_ and *σA*(*t*) for each group calculated over a period of 200 days post infection using $\overline{{\varepsilon }_{A}}=\frac{1}{200}{\int }_{0}^{200}(\frac{\eta A(t)}{1+\eta A(t)})dt$ and $\overline{\sigma A(t)}=\frac{1}{200}{\int }_{0}^{200}\sigma A(t)dt$. Note that for the rapid-progressor morphine group, we can obtain the analytic solutions as $\overline{{\varepsilon }_{A}}=\frac{\eta a}{1+\eta a}$ and $\overline{\sigma A(t)}=\sigma a$.

As revealed by our data fitting procedure, the main effects of virus specific antibody responses are the neutralization of virus and enhancement of viral clearance. Note that even though the median values of η and *σ* are similar in each group, the net effect of the antibodies related to the neutralization of virus and enhancement of viral clearance is determined by the combined terms ηA(t) and *σA*(*t*), respectively, which vary among groups. To quantify the effects due to morphine, we computed the efficacy of virus-specific antibodies in reducing the infection rate, ${{\epsilon }}_{A}$, and the rate of enhanced virus clearance, *σA*(*t*), for each group of animals. Our results at the end of the 200 day post infection period clearly show lower efficacy of antibody responses to reduce virus infection in the morphine-dependent groups when compared to the control group (${{\epsilon }}_{A}\approx 0$ for the rapid-progressor group, ${{\epsilon }}_{A}=5.0\times {10}^{-4}$ for the slow-progressor group, and ${{\epsilon }}_{A}=2.6\times {10}^{-3}$ for the control group (Fig. 4)). We also calculated the mean value of ${{\epsilon }}_{A}$ (Table 3) over the time course of 200 days post infection and found that the mean antibody neutralization efficacy was lower in the morphine groups than in the control group ($\overline{{\varepsilon }_{A}}=9.5\times {10}^{-7}$ for the rapid-progressor group, $\overline{{\varepsilon }_{A}}=2.4\times {10}^{-4}$ for the slow-progressor group, and $\overline{{\varepsilon }_{A}}=1.0\times {10}^{-3}$ for the control group). Similarly, we found that the rate of enhanced virus clearance for the 200 day post-infection period was lower in the morphine-dependent groups when compared to the control group (Approximately 0 day^−1^ for the rapid-progressor group, 2.0 day^−1^ for the slow-progressor group, and 6.1 day^−1^ for the control group (Fig. 4)). The mean value of *σA*(*t*) over the time course of 200 days post infection was also lower in the morphine groups when compared to the control group (Approximately 0 day^−1^ for the rapid-progressor group, 0.8 day^−1^ for the slow-progressor group, and 2.4 day^−1^ for the control group (Fig. 4)). These results suggest that morphine can alter the antibody responses resulting in substantial effects on virus dynamics.

### Effects of morphine-altered antibody response on CD4^+^ T cell count

**Figure 5:**
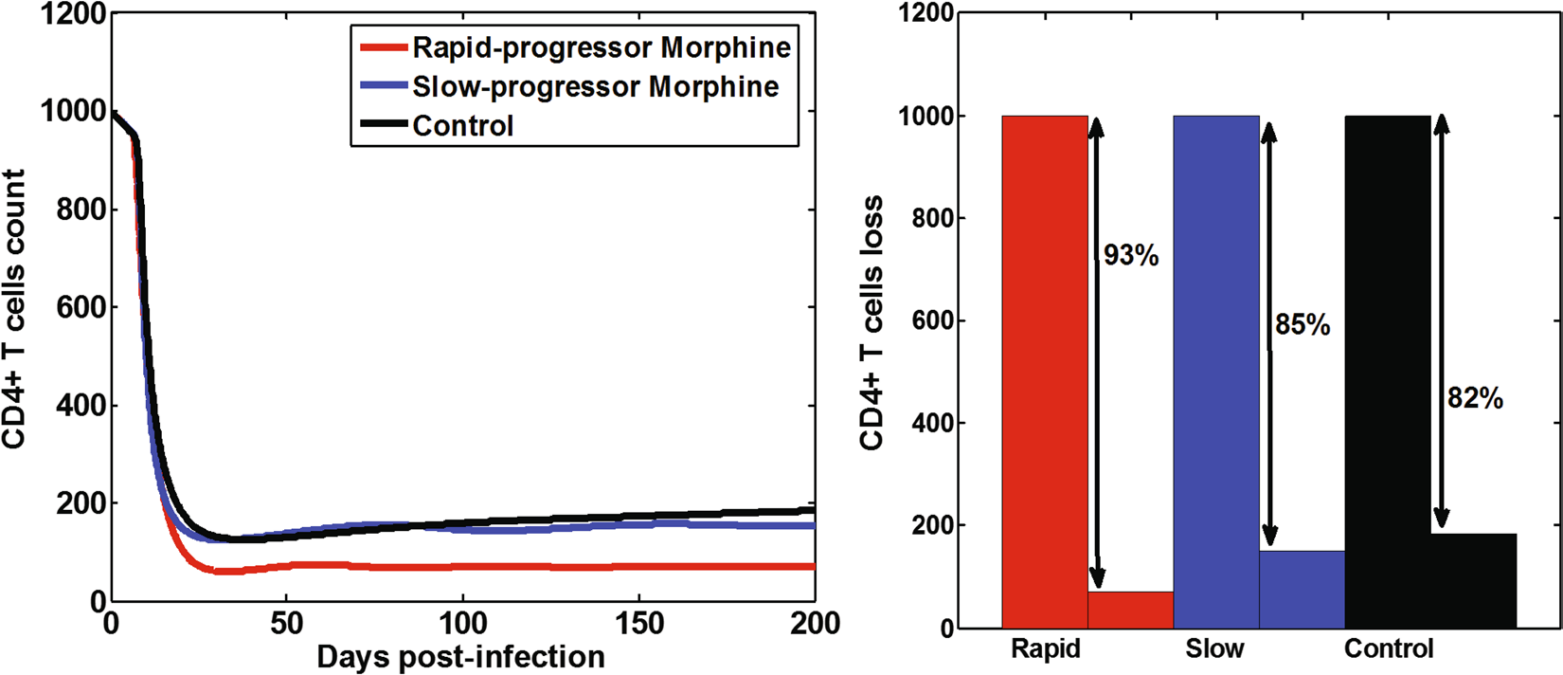
Predicted effects of morphine-altered virus-specific antibody responses on CD4^+^ T cells count and CD4 loss.

Using our model, we predicted the dynamics of the CD4^+^ T cell count (Fig. 5) and estimated the CD4^+^ T cell loss in the first 200 days post infection. The morphine group showed a lower CD4^+^ T cell count compared to the control group. At 200 days post-infection, we predict the CD4 count to be 70 cells/μL for the rapid-progressor morphine group and 152 cells/μL for the slow-progressor morphine group, while in the control group it is maintained at 185 cells/μL. With this prediction, we found a greater CD4^+^ T cell loss in the morphine-dependent groups (93% and 85% in the rapid-progressor and the slow-progressor morphine groups, respectively) than the control group (82% loss) (Fig. 5). We note that the CD4 data is more chaotic and not frequent enough to use for longer period fitting. However, we compared our results with the experimentally measured values of the CD4 count^8,18^, in which a loss of 99% at week 14 in the rapid-progressor morphine group, 97% loss at week 28 in the slow-progressor morphine group and 83% loss at week 28 of the control group were observed. This shows that our model predictions are qualitatively consistent with the experimentally measured values, but with a slight difference in magnitude.

### Basic reproduction number

We examine how morphine conditioning affects the basic reproduction number, *R*_0_, defined as the average number of secondary infections occurring from a single infected cell introduced into a population of entirely uninfected cells. *R*_0_ is an important measure of viral dynamics as it determines whether virus can establish infection. It can be shown that if *R*_0_ < 1, infection is avoided and if *R*_0_ > 1, infection is established^36^. With *A*(*t*) = *A*(0), we can derive the basic reproduction number of our model using the next-generation method^36^ as

$${R}_{0}=\frac{\lambda p({\beta }_{l}(d+q)+{\beta }_{h}r)(1-{{\epsilon }}_{A}(0))}{\delta d(d+r+q)(c+\sigma A(0))}.$$

Using our parameter estimates in this formula, we obtained ${R}_{0}^{R}=6.48$ (for the rapid-progressor morphine group), ${R}_{0}^{S}=2.12$ (for the slow-progressor morphine group), and ${R}_{0}^{C}=1.55$ (for the control group). Since *R*_0_ > 1 in all three groups, infection is predicted to occur in all the groups consistent with the data. Morphine does not seem to have role in determining establishment of the infection. However, having a higher value of *R*_0_ in the rapid-progressor morphine group indicates that the morphine may cause an extra obstacle that needs to be overcome in order to avoid infection by pre-exposure prophylaxis (PrEP). These estimated *R*_0_ values indicate that the effectiveness of PrEP required to prevent infection is at least 85% in the rapid-progressor morphine group, while 53% effectiveness and 36% effectiveness are needed to prevent infection in the slow-progressor morphine group and the control group, respectively.

## Discussion

HIV remains a major public health challenge and one of the highest causes of death worldwide, with a rapidly increasing dependency on drugs of abuse, such as opiates, in HIV-infected patients^5–7^. While drugs of abuse are known to affect HIV specific antibody responses^8^, how these alterations in antibody response impact within-host HIV dynamics is not well understood. Therefore, studying the effect of drugs of abuse on antibody responses and consequently on viral dynamics is importance. In this study, we extended our previous mathematical model of SIV dynamics under morphine conditioning^10^ by incorporating the effects of morphine-altered antibody responses. Our previous study that focused on viral dynamics for the first 3 months post-infection^10^ did not find any role of immune responses in virus dynamics, consistent with the low HIV-specific antibody levels during this period (Fig. 2). Here we studied viral dynamics for a longer period of time (Fig. 3) and found that the effect of morphine-altered antibody responses can become significant enough to alter long-term viral dynamics. This effect is particularly pronounced in a subpopulation of morphine-addicted animals, namely, a rapid-progressor morphine group, which exhibited faster disease progression resulting in a very short lifespan^8^. In another subgroup categorized as a slow-progressor morphine group^8^, which had a longer lifespan, the effect of the morphine-altered anitibody response was minimal. Our model provides useful insights into how morphine can affect virus-specific antibody responses and in turn SIV/HIV infection dynamics and disease progression.

Using our model and the data from SIV/SHIV infected rhesus macaques with and without morphine conditioning, we determined that the maximum antibody level (*a*) and the time when the antibody level becomes half-maximal (*b*) are significantly lower in the rapid-progressor morphine group than in the control group (Table 1, Fig. 2). Furthermore, excluding animal 1/52N, which has unusually high antibody levels and high CD4 counts, and using a two sample t-test with equal but unknown variances, these quantities also become significantly different between the rapid-progressor group and the slow-progressor group. Our results show that morphine has a significant effect on altering antibody responses, with a tendency to decrease virus-specific antibody levels and to cause a delay in the time to reach half-maximal antibody responses. Using these trends of the antibody response, our models further identified that the long term (200 days post infection) viral dynamics is best described by a model that includes two immune response effects: reduction of the cell infection rate and an increase in the virus clearance rate (Table 2). Our models do not support the third effect considered, namely, antibody-dependent infected cell killing.

Using the best supported model, we quantified the effect of morphine-altered antibody responses on the virus infection rate and the virus clearance rate for 200 days post-infection (Fig. 4). Our results show that the efficacy of antibody responses on reducing virus infection is significantly less in the morphine-dependent animals when compared to the control group (Fig. 4). Similarly, morphine dependence leads to less enhanced virus clearance in the slow-progressor morphine group and in the rapid-progressor morphine group than the control group (Fig. 4). A higher virus infection rate and/or a lower virus clearance rate in the morphine-dependent animals results in a higher viral load (Fig. 3). The dynamics predicted by the model also shows that CD4^+^ T cell count decreases faster in the morphine-dependent groups than in the control group. Thus, there is a higher CD4^+^ T cell drop in the presence of morphine (Fig. 5). Although this difference in CD4^+^ T cell drop was not statistically significant (p > 0.05), our results suggest that there are noticeable effects of morphine-altered virus-specific antibody responses on CD4 count and that morphine may exacerbate the disease progression.

We also computed the basic reproduction number, *R*_0_, as 6.48, 2.12, and 1.55 for the rapid-progressor morphine, the slow-progressor morphine, and the control groups, respectively, consistent with the observation that the infection got established in each group (*R*_0_ > 1). The higher value of *R*_0_ and the lower level of viral-specific antibody response in the rapid-progressor morphine group imply that morphine can make pre-infection intervention strategies, such as antibody-based vaccines and PrEP, less effective.

We acknowledge the several limitations of our study. Our model and related results are based on experimentally measured altered antibody responses due to morphine conditioning and does not address the mechanism by which morphine influences antibody production. However, to the best of our knowledge, the precise mechanism by which morphine alters virus-specific antibody responses is unknown and remains to be determined. Here a limited data set, including scarce antibody measurements, has been used to estimate parameters. Therefore, some of the numerical estimates may not be certain. More data from experimental studies including morphine conditioning and measured immune responses are needed to obtain more precise parameter estimates and related results. Based on successful estimates of the similar model parameters in the previous study^10^, in which even fewer data points of these animals were used, and the reasonable confidence intervals obtained for the estimated parameters in this study (Table 3), we expect that the parameters for our model are identifiable. We note that a detailed analysis of parameter indentifiability of our model may improve certainty of the estimated parameters. However, thorough identifiability analysis of this model might require sophisticated theoretical and computational methods. We considered only antibody responses in our model and observed that the viral dynamics is affected by the antibody responses. However, the effects on the viral dynamics may not be entirely due to the antibody levels and other immune responses such as those involving CD8^+^ cells or NK cells as well as weak engangement of viral antigens might have some effects on the viral dynamics. If multiple mechanisms were present to influence virologic outcomes, our results need to be interpreted carefully.

In summary, this study highlights the importance of virus-specific antibody responses on viral dynamics observed in SIV/SHIV infected animals and quantifies the effect of morphine-altered antibody responses on viral dynamics. Our analysis suggests that drugs of abuse may significantly diminish HIV-specific antibody responses, hence enhancing viral infection and disease progression in a subpopulation of HIV infected drug abusers. These results further suggest that alteration of immune responses due to the presence of drugs of abuse pose further obstacles to HIV control and prevention strategies, including development of antibody-based vaccines.

## Supplementary information

Supplementary Materials
