# Supplementary material for: Modeling the Effects of Morphine-Altered Virus Specific Antibody Responses on HIV/SIV Dynamics
Source: Sci Rep. 2019 Apr 1;9:5423. doi: 10.1038/s41598-019-41751-8 (PMC6443976; doi:10.1038/s41598-019-41751-8)
Supplement: Supplementary file 1 — Supplementary Materials [file 41598_2019_41751_MOESM1_ESM.pdf]

Supplementary materials for "Modeling the Effects of Morphine-Altered Virus Specific Antibody Responses on HIV/SIV Dynamics"

Jones M. Mutua[1], Alan S. Perelson[2], Anil Kumar[3], Naveen K. Vaidya[4, 5, 6, *]

[1] Department of Computer Science, Mathematics, and Physics, Missouri Western State University, St. Joseph, Missouri, USA

[2] Theoretical Biology and Biophysics Group, Los Alamos National Laboratory, Los Alamos, New Mexico, USA

[3] Division of Pharmacology, School of Pharmacy, University of Missouri – Kansas City, Missouri, USA

[4] Department of Mathematics and Statistics, San Diego State University, San Diego, California, USA

[5] Computational Science Research Center, San Diego State University, San Diego, California, USA

[6] Viral Information Institute, San Diego State Universtiy, San Diego, California, USA

* Corresponding author: nvaidya@sdsu.edu

Supplementary Figure S1. Fitted curves to virus-specific antibody levels from individual animals (the rapid-progressor morphine group: 1/04L, 1/28Q, 1/42N; the slow-progressor morphine group: 1/52N, 1/56L. 1/02N; the control group: 2/31P, 2/02P, 2/AC42, MAC-1, MAC-2, MAC-3. Note that for the rapid progressor morphine group, $A(t) = a = 0.50$, half of the ditection limit, was used (see main text for details).

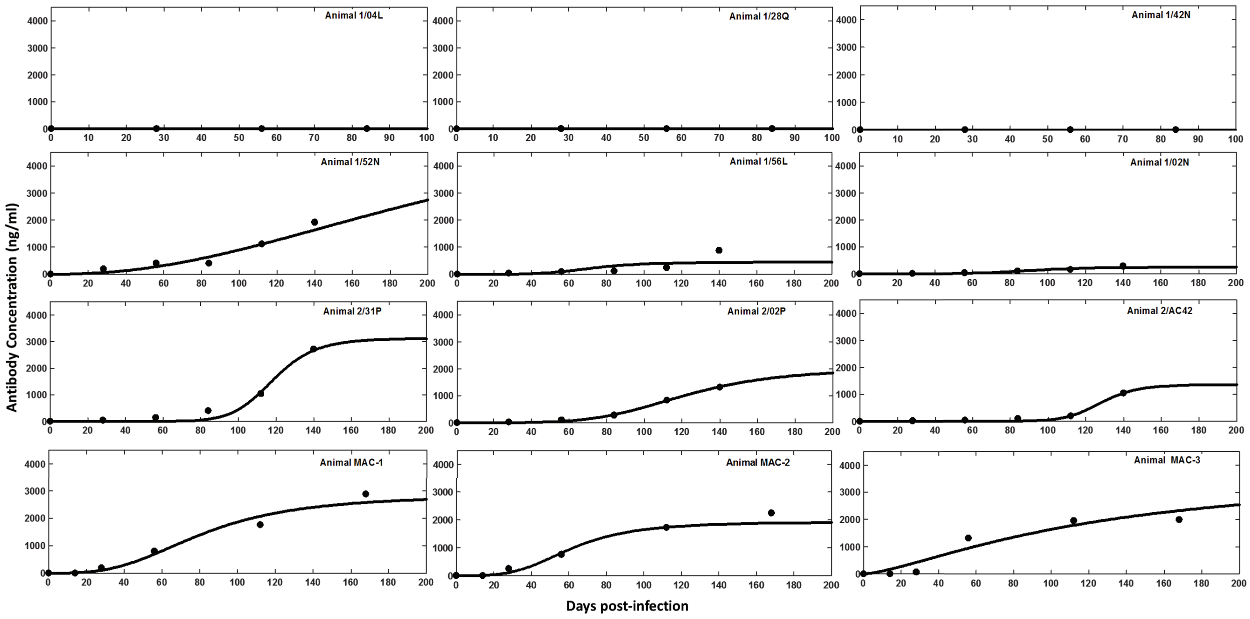

Supplementary Table S1. Model-1 estimated parameters for individual animals and their 95% confidence intervals in parentheses.

Animal	λ $(cell\ ml^{-1} day^{-1})$	r (day^{-1})	q (day^{-1})	η $(ml\ ng^{-1})$	γ $(ml\ ng^{-1} day^{-1})$	σ $(ml\ ng^{-1} day^{-1})$
**Rapid-progressor morphine group**						
1/04L	3630 (3311-3995)	0.22 (0.08-0.40)	$1.2x10^{-4}$ ($5.4x10^{-5}$ - $3.4x10^{-4}$)	$1.2x10^{-5}$ ($1.2x10^{-6}$ - $3.7x10^{-5}$)	$1.0x10^{-8}$ ($4.7x10^{-10}$ - $3.7x10^{-8}$)	$2.0x10^{-5}$ ($1.8x10^{-6}$ - $4.2x10^{-5}$)
1/28Q	3630 (3400-3870)	0.21 (0.07-0.45)	$1.5x10^{-5}$ ($4.1x10^{-4}$ - $3.4x10^{-4}$)	$1.4x10^{-5}$ ($5.1x10^{-6}$ - $3.2x10^{-5}$)	$1.0x10^{-8}$ ($1.6x10^{-9}$ - $3.6x10^{-8}$)	$1.3x10^{-5}$ ($4.7x10^{-6}$ - $3.9x10^{-5}$)
1/42N	3630 (3307-4021)	0.25 (0.18-0.84)	$1.6x10^{-4}$ ($6.3x10^{-5}$ - $3.0x10^{-4}$)	$1.0x10^{-5}$ ($6.5x10^{-6}$ - $2.7x10^{-5}$)	$1.1x10^{-8}$ ($6.5x10^{-9}$ - $3.5x10^{-8}$)	$1.1x10^{-5}$ ($5.0x10^{-6}$ - $3.8x10^{-5}$)
**Slow-progressor morphine group**						
1/52N	3629 (3628-3629)	0.29 (0.21-0.32)	0.25 ($0.19 - 0.29$)	$2.8x10^{-4}$ ($1.3x10^{-4}$ - $3.6x10^{-4}$)	$1.1x10^{-5}$ ($3.0x10^{-6}$ - $3.4x10^{-5}$)	$1.1x10^{-3}$ ($1.1x10^{-4}$ - $2.6x10^{-3}$)
1/56L	3630 (3580-3674)	0.22 (0.17-0.32)	0.20 ($0.21 - 0.29$)	$1.0x10^{-4}$ ($2.4x10^{-4}$ - $2.5x10^{-4}$)	$1.0x10^{-9}$ ($2.5x10^{-9}$ - $2.7x10^{-9}$)	$1.0x10^{-4}$ ($2.4x10^{-4}$ - $2.5x10^{-4}$)
1/02N	3630 (3629-3630)	0.21 (0.16-0.26)	0.20 ($0.15 - 0.25$)	$1.0x10^{-4}$ ($4.8x10^{-5}$ - $3.3x10^{-4}$)	$5.9x10^{-4}$ ($3.4x10^{-4}$ - $8.0x10^{-4}$)	$4.8x10^{-3}$ ($2.7x10^{-3}$ - $6.5x10^{-3}$)
**Control group**						
2/31P	3630 (3626-3633)	0.17 (0.13-0.20)	0.24 ($0.18 - 0.28$)	$1.3x10^{-4}$ ($7.9x10^{-5}$ - $1.8x10^{-4}$)	$1.0x10^{-9}$ ($1.1x10^{-10}$ - $2.4x10^{-9}$)	$1.0x10^{-4}$ ($3.1x10^{-6}$ - $2.2x10^{-4}$)
2/02P	3630 (3628-3630)	0.20 (0.13-0.22)	0.22 ($0.09 - 0.26$)	$2.0x10^{-4}$ ($1.2x10^{-5}$ - $2.0x10^{-4}$)	$9.8x10^{-9}$ ($4.6x10^{-9}$ - $1.6x10^{-8}$)	$2.0x10^{-4}$ ($7.4x10^{-6}$ - $2.0x10^{-4}$)
2/AC42	3630 (3613-3643)	0.20 (0.15-0.36)	0.34 ($0.33 - 0.45$)	$1.0x10^{-4}$ ($4.3x10^{-5}$ - $1.9x10^{-4}$)	$1.6x10^{-4}$ ($3.4x10^{-5}$ - $3.0x10^{-4}$)	$1.0x10^{-4}$ ($2.1x10^{-5}$ - $2.0x10^{-4}$)
MAC-1	3631 (3627-3635)	0.18 (0.13-0.30)	0.20 ($0.10 - 0.30$)	$1.0x10^{-4}$ ($1.5x10^{-5}$ - $2.8x10^{-4}$)	$1.0x10^{-9}$ ($2.9x10^{-10}$ - $2.7x10^{-9}$)	$1.0x10^{-4}$ ($5.2x10^{-6}$ - $3.5x10^{-4}$)
MAC-2	3630 (3629-3631)	0.14 (0.08-0.19)	0.20 ($0.08 - 0.26$)	$1.0x10^{-4}$ ($1.6x10^{-5}$ - $2.4x10^{-4}$)	$1.1x10^{-9}$ ($1.0x10^{-9}$ - $1.9x10^{-9}$)	$1.0x10^{-4}$ ($5.6x10^{-6}$ - $2.3x10^{-4}$)
MAC-3	3631 (3548-3694)	0.20 (0.20-0.42)	0.27 ($0.14 - 0.45$)	$1.0x10^{-4}$ ($7.8x10^{-6}$ - $3.5x10^{-4}$)	$1.0x10^{-9}$ ($2.3x10^{-10}$ - $2.8x10^{-9}$)	$1.0x10^{-4}$ ($9.3x10^{-6}$ - $3.7x10^{-4}$)

Supplementary Table S2. Model-2 (the best model) estimated parameters for individual animals, and the fitted parameter values to the group median data for $V_0 = 200$ (case when vRNA is dispersed in extracellular water) and V_0=1000 (case when vRNA is dispersed in blood plasma). The group median data represent the median data of each group.

Animal	V_0 (RNA copies/ml)	λ $(cell\ ml^{-1}day^{-1})$	r (day^{-1})	q (day^{-1})	δ (day^{-1})	σ $(ml\ ng^{-1}day^{-1})$	η $(ml\ ng^{-1})$
**Rapid-progressor morphine group**							
1/04L	200<br>1000	3630<br>3629	0.20<br>0.16	1.1×10^{-4}<br>1.3×10^{-4}	0.56<br>0.50	2.8×10^{-3}<br>1.1×10^{-3}	1.0×10^{-4}<br>1.1×10^{-5}
1/28Q	200<br>1000	3773<br>3630	0.24<br>0.22	1.0×10^{-4}<br>1.0×10^{-5}	0.80<br>0.72	2.3×10^{-3}<br>1.0×10^{-3}	1.1×10^{-4}<br>1.4×10^{-5}
1/42N	200<br>1000	5000<br>4701	0.40<br>0.31	1.2×10^{-4}<br>1.0×10^{-5}	0.30<br>0.29	2.3×10^{-3}<br>2.0×10^{-3}	1.1×10^{-4}<br>1.0×10^{-5}
Group median data	200<br>1000	3630<br>3630	0.16<br>0.15	1.0×10^{-2}<br>7.6×10^{-3}	0.31<br>0.30	1.1×10^{-2}<br>2.7×10^{-3}	1.9×10^{-6}<br>1.5×10^{-7}
**Slow-progressor morphine group**							
1/52N	200<br>1000	3631<br>3630	0.38<br>0.31	0.21<br>0.19	0.53<br>0.46	1.5×10^{-2}<br>1.2×10^{-3}	1.0×10^{-6}<br>1.3×10^{-6}
1/56L	200<br>1000	3629<br>3630	0.41<br>0.36	0.18<br>0.10	0.32<br>0.30	2.3×10^{-6}<br>1.0×10^{-6}	1.0×10^{-6}<br>3.1×10^{-6}
1/02N	200<br>1000	3630<br>3630	0.18<br>0.15	0.10<br>0.08	0.75<br>0.66	3.0×10^{-2}<br>2.5×10^{-2}	1.1×10^{-6}<br>1.0×10^{-7}
Group median data	200<br>1000	3629<br>3630	0.17<br>0.10	0.10<br>0.10	0.65<br>0.50	3.4×10^{-3}<br>2.0×10^{-3}	1.0×10^{-6}<br>1.0×10^{-7}
**Control group**							
2/31P	200<br>1000	3629<br>3630	0.31<br>0.26	0.68<br>0.59	0.31<br>0.28	7.1×10^{-3}<br>1.0×10^{-4}	1.0×10^{-6}<br>1.0×10^{-7}
2/02P	200<br>1000	4050<br>3700	0.18<br>0.16	0.20<br>0.11	0.66<br>0.60	2.3×10^{-3}<br>1.1×10^{-4}	1.0×10^{-4}<br>2.6×10^{-5}
2/AC42	200<br>1000	3630<br>3630	0.14<br>0.11	0.33<br>0.29	0.54<br>0.53	5.3×10^{-4}<br>2.1×10^{-5}	4.9×10^{-5}<br>1.1×10^{-6}
MAC-1	200<br>1000	3630<br>3630	0.13<br>0.10	0.18<br>0.11	0.38<br>0.36	2.3×10^{-3}<br>2.0×10^{-4}	1.0×10^{-4}<br>1.4×10^{-5}
MAC-2	200<br>1000	3629<br>3630	0.13<br>0.12	0.28<br>0.24	0.61<br>0.58	3.2×10^{-6}<br>1.8×10^{-6}	1.0×10^{-7}<br>1.1×10^{-7}
MAC-3	200<br>1000	3630<br>3630	0.16<br>0.11	0.24<br>0.21	0.40<br>0.36	2.3×10^{-6}<br>1.0×10^{-7}	1.0×10^{-6}<br>1.0×10^{-7}
Group median data	200<br>1000	3630<br>3630	0.15<br>0.14	0.18<br>0.17	0.65<br>0.65	2.5×10^{-3}<br>1.0×10^{-4}	1.1×10^{-6}<br>1.1×10^{-7}
